# Provision of pharmaceutical care to suspected high-risk COVID-19 patients through telehealth: a nationwide simulated patient study

**DOI:** 10.1186/s12913-021-07014-x

**Published:** 2021-09-21

**Authors:** Rania Itani, Hani M J Khojah, Fatima Jaffal, Deema Rahme, Lina Karout, Samar Karout

**Affiliations:** 1 Pharmacy Practice Department, Faculty of Pharmacy, Beirut Arab University, Riad El Solh, P.O. Box: 11- 5020, 1107 2809 Beirut, Lebanon; 2 Department of Clinical and Hospital Pharmacy, College of Pharmacy, Taibah University, P.O. Box 30051, 41477 Madinah, Kingdom of Saudi Arabia; 3 Department of Radiology, American University of Beirut Medical Center, Riad El-Solh, P.O. Box: 11-0236, 1107 2020 Beirut, Lebanon

**Keywords:** COVID-19, Community pharmacy, Telehealth, Pharmaceutical care, Lebanon

## Abstract

**Background:**

The COVID-19 pandemic has overburdened the healthcare facilities, which demanded the use of alternative and effective methods for delivering healthcare services. The use of telehealth has become a necessity to provide initial health services.

**Objective:**

To identify the pharmaceutical care provided by community pharmacists to suspected high-risk COVID-19 patients using telehealth.

**Methods:**

A simulated patient (SP) phoned 100 randomly-selected community pharmacies throughout Lebanon using a standard scenario of uncontrolled diabetes mellitus with typical symptoms of COVID-19. Pharmacists’ responses were compared with pre-defined ideal recommendations using a special form.

**Results:**

The mean of the retrieved medical information score obtained by the pharmacists was 2.48 ± 2.79 (out of 21), with 34 % of the participants not retrieving any relevant medical data from the SP. The relative patient information, the exposure to COVID-19, and the possible COVID-19 symptoms were not retrieved by 61 %, 70 %, and 41 % of the pharmacists, respectively. Two percent of the pharmacists assured that the SP’s symptoms were related to common cold, while 5 % confirmed that the SP is infected with COVID-19. Notably, 35 % of the pharmacists did not offer any recommendation. Among them, 14 % claimed that they were too busy to respond. Only 39 % of the pharmacists provided an appropriate recommendation by referring the SP to her physician to seek medical attention within 24 h since the SP is a high-risk patient, and 41 % recommended doing a PCR test. Antipyretics, antibiotics, and dietary supplements were recommended by 27 %, 7 %, and 16 % of the pharmacists, respectively. Less than 16 % of the pharmacists recommended using protective measures against COVID-19. In addition, the overall communication skills of the pharmacists were generally below expectations.

**Conclusions:**

This study is the first to assess the quality of pharmaceutical care provided by community pharmacists in the Middle East via Telehealth. An unsatisfactory level of preparedness through means of telehealth technology was evident. This resulted in the quality of pharmaceutical-care services provided to high-risk patients via telehealth to be below expectations. Therefore, health authorities should encourage community pharmacists to effectively adopt telehealth, by providing appropriate training, as well as recognizing their extra efforts with financial compensations, aiming to optimize patients’ health outcomes.

**Supplementary Information:**

The online version contains supplementary material available at 10.1186/s12913-021-07014-x.

## Introduction

The COVID-19 pandemic has stretched the capacity of the healthcare systems, increasing demand for life-saving medications, revealing disturbing barriers in patient care access, and exacerbating the shortage of healthcare professionals [1, 2]. This crisis has placed tremendous pressure on the frontline medical teams, stimulating cooperation among different medical professionals to combat the pandemic [3, 4]. Over the years, pharmacists have been working hard to prove and establish themselves as core members in the provision of patient care, and today, amid the pandemic, they have had the opportunity to effectively embrace their role as frontline healthcare providers shifting the concept of the pharmacy profession [5, 6]. Apart from their initial patient-centered role, community pharmacists were involved in controlling the SAR-COV-2 virus transmission, assessing potential COVID-19 carriers, and triaging suspected cases [5, 7, 8].

By December 2020, the Lebanese healthcare system became saturated as hospitals were unable to serve all COVID-19 patients who required medical care [9]. Community pharmacists were involved in providing medical services for suspected COVID-19 patients, especially because there were limited resources available for free screening offered by the government [2, 5, 8]. Many patients primarily sought and continue seeking medical advice from community pharmacists, who provided consultations free of charge. Besides, most symptomatic patients amenable to self-treatment sought help from community pharmacists on non-prescription drugs [5]. As providers of pharmaceutical care and free consultations, pharmacists were being placed under extreme pressure to meet patients’ demands, escalating both patients’ and pharmacists’ risks of contracting the infection [2]. As a result, community pharmacists had to implement various infection control strategies into their daily practice in pharmacy operations aiming to contain the spread of COVID-19 [10–14]. Consequently, shifting to a more convenient, accessible, and cost-effective method of communication to deliver healthcare services was necessary, such as telehealth.

By definition, telehealth is the delivery of health-related services by medical professionals through distance, via digital communication technologies [15]. Telehealth services were at the center of the medical field’s response to this pandemic, particularly when patients were in quarantine [16]. Moreover, medical providers have shown a willingness to use telehealth systems to continue offering health assistance during the pandemic [17, 18]. The potential benefit of telehealth in minimizing the transmission of COVID-19 in primary healthcare facilities comes into sharp focus when patients with respiratory symptoms start seeking medical attention from community pharmacies, especially when hospitals became unable to meet their demand [1, 19]. Furthermore, it helped in providing clinical services and triaging patients, so that other healthcare professionals were not overwhelmed with new presentations [20]. Providing valuable health services and communication by telehealth is crucial for elderly individuals and those with underlying chronic medical conditions, such as diabetes mellitus (DM), hypertension, and other cardiovascular diseases. These patients are more susceptible to develop more severe illnesses once infected with the SARS-CoV-2 virus. Findings from recent studies have shown that there was a two-fold increase in the severity of COVID-19 cases among patients with DM. In addition, a significantly higher mortality rate was observed among COVID-19 patients having DM [21–23].

Prior to the COVID-19 pandemic, the use of telehealth in Lebanon was limited, although smartphones were largely available, and the internet was easily accessible in the country [24]. Nonetheless, telehealth services have been increasingly adopted during the current situation, so they have become an effective integral component across the healthcare landscape [25, 26]. The literature review did not reflect any study assessing telehealth services provided by community pharmacists in the Middle East, especially during the pandemic. Therefore, the present situation has raised concerns about the preparedness of community pharmacists to adopt telehealth technology. At the time of writing this manuscript, most studies that evaluated the implementation of telehealth services were self-administered questionnaires prone to bias and did not reflect the actual behavior of pharmacy staff [27, 28].

Hence, this study aimed to identify the community pharmacists’ preparedness in response to the COVID-19 outbreak through the implementation of telehealth services, specifically tackling suspected high-risk COVID-19 patients.

## Materials and methods

### Study design

This study was conducted during January 2021, using an unrevealed simulated patient (SP), a technique that recently gained popularity in research assessing pharmaceutical care services in developing countries. This method has considerable advantages because it limits the Hawthorne Effect, in which the study subjects modify their behavior when they are aware of being observed [29]. Therefore, this study will genuinely reflect the actual quality of performance and services provided in the pharmacies.

A female researcher was trained to play the role of a simulated patient, following a well-structured scenario to identify the actual and spontaneous behavior of the community pharmacists. The SP is one of the study authors, so no further ethical parameters were required. The SP acted as a patient with uncontrolled diabetes mellitus who was complaining of typical symptoms of COVID-19, including fever, dry cough, and fatigue. A recent systematic review and meta-analysis have estimated the ‎prevalence of the clinical symptoms of COVID-19, revealing that fever (81.2 %), cough ‎‎ (58.5 %), fatigue (38.5 %), and dyspnea (26.1 %) are the most commonly associated symptoms. ‎ Considering those main symptoms is crucial for early detection of COVID-19 and ‎prevention of its transmission [30]. Further information regarding the SP’s medical condition was provided only upon request by the pharmacists. Finally, the SP documented each pharmacist’s responses using a data collection form, either during or immediately after the call to minimize any possible recall bias.

### Selection of pharmacies

The community pharmacies in Lebanon consist of independent retail pharmacies, privately owned by registered Lebanese pharmacists; and are the only authorized source for dispensing medications to the public [31]. Currently, there are 2,897 community pharmacies operating all over Lebanon, distributed over the five main districts (Beirut, North, South, Mount Lebanon, and Bekaa). This study was carried out using a proportionate random sample of community pharmacies from each district. A list of registered community pharmacies was obtained from the Lebanese Order of Pharmacists’ website (LOP) [32]. The list included the commerce names of community pharmacies, the name of each pharmacy’s owner, along with the designated district, address, and phone number. Then, the pharmacies were sorted per district, and Microsoft Excel was utilized to randomly shuffle the ‎pharmacies after coding them, using the RAND function, ‎ to obtain an equal number of pharmacies from each district.

The online Raosoft^®^ calculator was used to determine the sample size for the current study [33]. It was assumed that 50 % of the Lebanese pharmacies (out of 2,897) would provide appropriate pharmaceutical care for patients with suspected COVID-19 through telehealth. The sample size was calculated at a 95 % confidence interval using an absolute precision of 10 %, and a design effect of 1 since we used a proportional random sampling. Thus, the calculated sample size for the current study to achieve a representative sample was 100, in which we randomly selected 20 pharmacies from each district ‎(Beirut, North, South, Mount Lebanon, and Bekaa). ‎A pharmacy was replaced with the next one from the remaining shuffled list, within the same district, whenever the call was not answered upon three spaced attempts during the study period. Each pharmacy that responded to the call was contacted only once.

### Simulated-patient scenario

The SP called the community pharmacy seeking consultation from the pharmacist about her symptoms. She stated that she has DM and seemed worried about her fever. Her DM is uncontrolled, and her blood glucose level is fluctuating, where the fasting blood glucose (FBG) ranged between 140 and 160 mg/dl, and random blood glucose (RBG) was between 170 and 210 mg/dl. Afterward, the SP asked the pharmacist what she should do in this regard.

The following information was provided only upon the pharmacists’ request. The SP was a single, unemployed 30-year-old female who lived with her 70-year-old mother who suffered from hypertension and congestive heart failure. The SP was diagnosed with type 1 diabetes mellitus (T1DM) for 10 years, for which she was taking Lantus® (insulin glargine), 30 units at bedtime, and Apidra Solostar® (insulin glulisine), 10 units 15 min before each meal. The SP’s diabetic status was previously controlled on these insulin doses for the last 6 months. The SP’s last medical visit to her physician was 2 months ago. She did not follow a certain diet and only avoided direct sugar intake. She had a sedentary lifestyle with no time to exercise. She had several symptoms over the last 2 days, including fever (38–39 °C), generalized fatigue, body aches, dry cough, anosmia, and dizziness. The SP denied the presence of any COVID-19 red-flag symptoms that require emergent medical attention, such as severe dyspnea, new confusion, pale or blue-colored skin, or severe chest pain [34]. When asked about the medications taken to cope with the current symptoms, she reported that she was taking Panadol® (paracetamol or acetaminophen), 2 tablets 3 times daily without any noticed improvement. The SP also claimed that she had not traveled recently, had not attended any social gatherings, and that she did not know if she had recently been in contact with a COVID-19 case. She had not undergone the polymerase chain reaction (PCR) test before, and she was not in isolation at the time of the phone call.

### Data collection form development and structure

The research team developed a data collection form based on the “Phone Advice Line Tool” that was issued by the Centers for Disease Control and Prevention (CDC), which guides the healthcare practitioners to advise possible COVID-19 patients on seeking appropriate medical care, through the telehealth communication services [34]. The data collection form includes screening for COVID-19 exposure and symptoms, evaluating life-threatening conditions, assessing high-risk patients, a decision algorithm for appropriate case disposition, and tailored care advice messages.

The form included checkboxes, predefined choices, and was composed of four sections. The first section included the characteristics of the contacted pharmacy, such as its location/district (according to the LOP directory), code, and the pharmacist’s sex. The second section collected the relevant data retrieved by the contacted pharmacist. This section was further divided into subcategories, which included patient-related information (e.g., SP’s age, occupation, residence, FBG/RBG readings, and chronic medical conditions/medications), COVID-19 exposure assessment (close contact with COVID-19 cases, travel history, and attendance in crowded social gatherings), and COVID-19-related symptoms and treatments. The third section documented the pharmacist’s responses and recommendations provided to the patient to manage her condition, which included pre-specified options as checkboxes and open-ended answers to record the participants verbatim.

Finally, the last section described the pharmacist’s general communication skills throughout the call. This section was composed of a simple rubric, developed by the study authors, that recorded the presence or absence of the following items; “‎Introduced him/herself ”, ‎“Solicited the patient’s agenda”, “Used proper and clear language without jargon terminologies”, “Showed empathy ‎(demonstrated a sincere interest in the patient’s emotional needs)‎”, “Demonstrated active listening (provided feedback, briefly summarized ‎his/her understating to clarify any message), ‎and “‎Gave appropriate closure (summarized the patient’s concern and asked if ‎she had any other ‎questions)‏”.

The form was then introduced to two academic researchers in the field of pharmacy surveys, and two ‏professional experts running their community pharmacies. The experts reviewed the form and suggested ‏adding additional data related to diabetes status and symptoms that should be retrieved by the participants ‏to assess the SP’s condition. Amendment to the form was done accordingly (Additional file 1).

### The pilot test

Before the commencement of the study, the protocol was tested on fifteen pharmacies aside from the randomly selected ones, where the SP called the pharmacies in the presence of another researcher. This aided in testing the simulation, assessing the scenario’s feasibility, and ensuring the reliability and reproducibility of the data collection form.

### Operational definitions

#### The retrieved medical information score

A scoring system was developed to measure the degree of relevant medical information retrieved by the pharmacists. The items included in this scoring system are mainly recommended by the CDC’s “Phone Advice Line Tool”, which comprises a patient’s information and medical status regarding the high-risk condition (DM), history of COVID-19 exposure, and COVID-19 related symptoms and treatments [34] (Table 1). The score ranged from 0 to 21, where one mark was counted for each piece of relevant medical information retrieved.

**Table 1:** Relevant medical information requested by the pharmacists

**A. Patient’s information and medical history regarding DM**	n (or %)^*^
Age	12
Pregnancy/lactation	0
Duration of DM	0
FBG and RBG readings	33
Frequency of routine DM clinic visits	0
Other chronic medical condition(s)	0
Chronic medication(s)	22
Adherence to diet and physical activity	20
Controlled/uncontrolled DM before the onset of new symptoms	17
Presence of fruity-smelling breath	0
Constant/fluctuating insulin doses before the onset of new symptoms	0
*None of the above was obtained by the pharmacist*	*61*
**B. Assessment of COVID-19 exposure**	
Occupation (to rule out high-risk occupations such as HCP and LTCF personnel)	4
Residence and members sharing living	6
Close contact with someone with symptoms, or diagnosed or tested positive for COVID-19 within 2 weeks before feeling sick	29
Travel history	0
Attending a social gathering, or being in crowded indoor settings	7
*None of the above was obtained by the pharmacist*	*70*
**C. Assessment of COVID-19-related symptoms and treatments**	
Symptoms of COVID-19 (e.g., cough, shortness of breath or difficulty breathing, fever or chills, muscle or body aches, recent loss of taste or smell, vomiting or diarrhea)	57
Onset and duration of symptoms	21
Life-threatening symptoms	7
Medications used to manage symptoms	10
History of flu vaccination	3
*None of the above was obtained by the pharmacist*	*41*

*DM* diabetes mellitus, *FBG* fasting blood glucose, *HCP* health care provider, *LTCF* long-term care facility, *RBG* random blood glucose

^*^Since the number of pharmacists is 100, the result indicates the percentage too. As mixed responses were given, numbers do add up to 100

#### Appropriate recommendation

According to the CDC’s “Phone Advice Line Tool”, a patient complaining of typical COVID-19 symptoms, with no life-threatening symptoms, and belonging to a high-risk condition (diabetes mellitus in this case), should be recommended to refer to his/her physician within 24 h [34].

### Data analysis

Data were analyzed using the IBM *Statistical Package for the Social Sciences* (*SPSS*®) software version 24. The descriptive data were represented using frequencies and percentages for categorical variables, and mean with standard deviation for continuous variables. The retrieved medical information score‎ was tested for normality using Shapiro-Wilk tests before statistical ‎comparisons. In addition, the association between the “retrieved medical information score” with the pharmacists’ gender and designated district of the pharmacy was tested by Kruskal-Wallis and Mann-Whitney U tests. The association between the bivariate “appropriate recommendation” (Yes/No) with independent variables was tested using the Pearson Chi-square (χ^2^). The significance level was set at *P* ≤ 0.05 with a confidence interval (CI) of 95 %.‎.

### Ethical considerations

The guidance of the World Medical Association Declaration of Helsinki was followed in designing ‎and ‎‎conducting this study. ‎The study protocol was approved by the Institutional Review Board of Beirut Arab University (No. 2021-H-0073-P-R-0441) with the waiver of the informed consent, since it was impractical to obtain it from the participants due to potential alteration in their behavior. The contacted pharmacists’ confidentiality and anonymity were maintained by several procedures. The database was password-protected by a colleague who was not among the research team, and the details of the pharmacy and pharmacist identification were replaced by codes. Pharmacies were not contacted afterward for additional questioning.

## Results

A total of 100 community pharmacies were phoned by the SP for the purpose of the present study. Two pharmacies had to be replaced because of a busy line despite three separate calls. The distribution of the pharmacists’ genders was almost equivalent, as 55 % were males and 45 % were females. The duration of phone calls was 1.85 ± 1.13 min (range = 0.5–8).

### Relevant medical information requested by the contacted pharmacists

The mean of the retrieved medical information score obtained by the pharmacists was 2.48 ± 2.79 (out of 21), with 34 % of the participants not retrieving any relevant medical data from the SP. As reflected in Table 1 A, the majority (61 %) of the pharmacists did not request any information related to DM, and only 33 % were concerned about obtaining the FBG and RBG levels from the SP. In addition, no pharmacist asked about other critical information, like the consistency of insulin doses before the onset of the new symptoms, or the presence of fruity-smelling breath that indicates severe hyperglycemia.

A similar response was noted regarding the assessment of possible SP’s exposure to COVID-19 (Table 1B), where 70 % of the pharmacists were not concerned about obtaining any relevant information in this regard. In addition, only 29 % of the pharmacists showed some degree of concern. However, a slightly better outcome was observed for the assessment of COVID-19 symptoms and symptomatic treatments since 3–57 % of the pharmacists demonstrated various positive responses. Nevertheless, 41 % of the pharmacists retrieved neither symptoms nor treatment-related information from the SP, as shown in Table 1 C. The retrieved medical information score was not significantly different between males (mean = 2.31 ± 2.88) and females (2.69 ± 2.71, *P* = 0.31). Similarly, the score was not significantly different among pharmacies in various districts (*P* = 0.33).

### Pharmacists’ responses and recommendations

Two pharmacists (2 %) assured the SP that her symptoms were related to the common cold, “Don’t worry you don’t have COVID-19, this is just a common cold” (Pharmacist 27). On the other hand, 5 % confirmed that the SP was infected with COVID-19, “You are definitely infected with COVID-19” (Pharmacist 39). In addition, 2–27 % of the pharmacists recommended some treatments to overcome the symptoms. These included antipyretics (paracetamol and ibuprofen), antiplatelets (aspirin), antibiotics (azithromycin), vitamins (multivitamins, vitamin C, and vitamin D), and supplements (Zinc). Moreover, 2–15 % of the pharmacists recommended various protective measures, such as isolation, social distancing, and the use of personal protective equipment. Only 39 % of the pharmacists referred the SP to her physician to seek medical attention within 24 h since the SP is a high-risk patient, and 41 % recommended doing a PCR test. Details about these results are depicted in Table 2. Moreover, there was no significant difference in the frequency of recommending seeking medical advice among the sexes (*P* = 0.21), and districts (*P* = 0.54).

**Table 2:** Pharmacist responses against the suspected COVID-19 case

Responses	n (or %)^*^
**Pharmacists’ responses related to possible COVID-19 infection**	
* Assured the SP that her symptoms are related to the common flu*	2
* Informed the SP that she might have been infected with COVID-19*	38
* Confirmed that the symptoms are related to COVID-19*	5
* Recommended an antipyretic for fever*	27
* Recommended other medication(s)*	7
* Recommended a supplement*	16
* Recommended non-pharmacological measure(s)*	2
* Advised the SP to adhere to PPE (e.g., facial mask, hand disinfectant, and gloves)*	6
* Advised the SP to monitor red flag symptoms*	11
* Advised the SP to isolate herself for 14 days*	4
* Advised the SP to avoid contacting others (especially her mother)*	15
* Advised the SP to seek medical attention because she was a high-risk patient*	39
* Advised the SP to test for PCR*	41
* Advised the SP to consult a health care provider if the PCR test was positive*	3
* Advised the SP to contact the Ministry of Public Health*	2
**Recommendations related to DM**	
* Advised the SP to keep monitoring her blood glucose levels*	16
* Advised the SP to avoid sugar intake*	11
* Advised the SP to increase her insulin dose*	2
* Advised the SP to check the HbA1C level*	2
**No recommendations were given**	
* The pharmacists stated that he/she was busy and requested ending the call*	14
* The pharmacists did not give any recommendation and advised the SP to seek medical attention*	20
* Advised the SP to consult a physician because pharmacists are not qualified to handle such a case*	1

*DM *diabetes mellitus, *Hb* hemoglobin, *PCR* polymerase chain reaction, *PPE* personal protective equipment, *SP* simulated patient

^*^Since the number of pharmacists is 100, the result indicates the percentage too. As mixed responses were given, numbers do add up to 100

Furthermore, only 2–16 % of these community pharmacists offered different consultations regarding the symptoms related to DM. Remarkably, 35 % of the pharmacists gave no recommendations at all. Among them, 14 % claimed they were too busy to respond, “Sorry I’m busy and I have to end the call” (Pharmacist 23). Surprisingly, one pharmacist stated that pharmacists are not qualified to handle such cases, “Why are you contacting me? It is not our duty to help in such a situation” (Pharmacist 15).

### Pharmacists’ communication skills

A significant number of pharmacists introduced themselves to the patient (87 %), used proper and clear language with no jargon (78 %), and solicited the patient’s agenda (73 %). More than half of the contacted pharmacists gave appropriate closure and summarized the patient’s concern (56 %), and 49 % showed empathy and sincere interest in the patient’s emotional needs. A lower number demonstrated active listening (38 %) by briefly summarizing the patient’s concerns and provided feedback.

## Discussion

To our knowledge, this is the first study that has highlighted the role of community pharmacists in the Middle East who have provided healthcare services using telehealth communication to suspected COVID-19 patients. The drastic rise in the number of confirmed and suspected COVID-19 patients has led to logistic dilemmas and has burdened hospitals and medical equipment supply chains [35, 36]. Ensuring the continuity of care and compliance with appropriate dispensing practice has become more complex, which escalates the importance of telehealth as a strategy that could aid in mitigating the interference of COVID-19 in the provision of pharmaceutical services. Even though the conception of telehealth is not novel, transitioning to this method of communication as an alternative to traditional approaches has been scaling up in medical practice settings during the COVID-19 pandemic. This is because telehealth services are thought to be an efficient way for providing remote access to quality healthcare services without increasing the risk of virus transmission, from and to, both healthcare providers and patients [37].

The CDC has issued guidance for health care professionals to respond to patients complaining of COVID-19 symptoms and seeking appropriate medical care through telehealth communication services [34]. The simulated scenario of the current study included a patient with diabetes mellitus, who was more susceptible to acquiring COVID-19. This high-risk patient complained of fever and general fatigue. The CDC guidance for such a case instructs the healthcare providers to ask the patient about the age, life-threatening symptoms, exposure to COVID-19, and COVID-19 related symptoms. The medical data obtained from the suspected cases assist in categorizing patients and affect the decision and medical advice of the healthcare providers. However, our findings revealed that only 12 % of the respondents asked about the patient’s age, 7 % inquired about the presence of life-threatening symptoms, and 57 % asked about the nature of the symptoms, while the exposure to COVID-19 was not assessed by most respondents (70 %). The mean of the retrieved medical information score was markedly low (2.48 ± 2.79), which might raise concerns on the quality of pharmaceutical services provided to patients, particularly through the use of tele-pharmacy services. This finding indicates that many community pharmacists are not ready to embrace new technology services such as telehealth. A cross-sectional survey study conducted in Jordan has shown that many pharmacists are doubtful to provide non-face-to-face services unless their healthcare outcomes become evident [38]. Another cross-sectional study conducted in the Netherlands found very limited use of telehealth services in educating patients about COVID-19, and a very large proportion was hesitant in the provision of quality services through telecommunication for vulnerable people with health issues [39]. However, Liu et al., have demonstrated positive effects in the implementation of remote pharmacy services in reducing patient crowding, and in decreasing the risk of cross-infection during pharmacy visits [40]. Furthermore, New Zealand and Australia have issued hotline numbers to encourage phone consultations during the pandemic [41, 42].

The quality of patient counseling regarding DM, its related symptoms, and treatment was also below expectations. This may be, in part, due to the lack of time pharmacists had because of their large number of contacts and clients during the outbreak in Lebanon. This was supported by our findings that 14 % of the pharmacists requested ending the call because they were busy and unable to handle consultations through the phone. In addition, the CDC has also recommended advising patients with high-risk conditions, including DM, to seek medical attention from their healthcare provider within 24 h [34]. This appropriate referral was provided by only 39 % of the pharmacists. It should be emphasized that community pharmacists have an indispensable role in containing the pandemic, where they are involved in properly screening patients and referring those with suspected COVID-19 to appropriate medical facilities in a timely manner [43]. Thus, increasing the accessibility for triaging and referral of more serious cases are both crucial to flatten the curve and optimize patient’s health outcomes. In addition, only 41 % of them considered advising the patient to undergo a PCR test. The pharmacists may have contemplated the PCR test primarily as a confirmatory test since the overlapping of symptoms between COVID-19 and other viral infections restrains their ability to confirm that it is a COVID-19 case. In contrast, 5 % of the pharmacists confirmed that the patient was infected with COVID-19, while 2 % assured the patient that her symptoms were related to the flu. This analysis necessitates a further discussion on the previously-mentioned information that symptoms support the diagnosis, yet do not confirm it. Community pharmacists have an important role in containing the COVID-19 pandemic in collaboration with other healthcare providers. However, 1 % of the pharmacists, in the present study, denied being qualified to handle a suspected COVID-19 case. Pharmacists around the world are involved in assessing COVID-19 patients and possible carriers, aiding in out-patient management, triaging high-risk patients or those who complain of any COVID-19 red flags, and providing patient care by dispensing an appropriate medication and disease counseling [5, 7, 8]. Moreover, pharmacists should aim to educate such patients to adhere to PPE and to avoid contacting others. However, these recommendations were only adopted by 6 and 15 % of our sample of community pharmacists, respectively. Moreover, there was an unexpected behavior in which 7 % of the pharmacists prescribed antiplatelets and/or antibiotics to the SP, violating the laws and regulations of pharmacies and pharmaceuticals issued by the Lebanese Ministry of Public Health (MOPH) [44]. Community pharmacists in Lebanon do not have the right to dispense any medicine that is not requested through a unified prescription unless the medicine is considered an over-the-counter (OTC) drug mentioned on the OTC list issued by the Lebanese MOPH [45]. In addition to the time barrier mentioned earlier, other challenges may have contributed to the unprofessional behaviors observed in this study. These may include the lack of financial return from the telephone consultations and the lack of training available for community pharmacists to utilize different telehealth systems. In contrast, in New Zealand, the government has recognized pharmacists’ extra efforts during the pandemic by the provision of extra remuneration for their support [42]. Furthermore, a Japanese study showed that a large number of healthcare providers (HCPs) considered financial incentives as an essential factor in their willingness and motivation to continue their long-term battle in COVID-19-related work [46]. Therefore, strategies aiming to address these challenges can involve reimbursements and appropriate guidance and training plans to ensure the execution of comprehensive and uniform telehealth services by pharmacists.

A high level of communication skills by a pharmacist during a phone call is essential to compensate for the lack of visual cues. Accordingly, the contacted pharmacist should introduce himself/herself, solicit the patient’s agenda, use proper and clear language throughout the call, show empathy, demonstrate active listening, and give an appropriate closure. The current study, however, has shown inconsistent responses by the community pharmacists through telehealth. Although around three-quarters of them introduced themselves, used proper language and gave the SP the chance to pose her problems, only half of them showed an acceptable level of empathy and provided a proper closure technique. Moreover, active listening was observed with as low as 38 % of them. Empathy is crucial to build rapport with the patient and gain his/her trust leading to improved health outcomes [47]. Furthermore, active listening falls beyond the physical process of hearing and comprises three components to comprehend, retain the key points, and respond [48]. Therefore, it is highly recommended that community pharmacies use the interactive communication loop with patients seeking medical advice through telehealth services, where the teach-back method ensures the understanding of both the listener and the speaker. Finally, we recommend future studies to evaluate the effect of telehealth on patients’ health outcomes and evaluate the cost-effectiveness of this healthcare paradigm.

### Limitations of the study

Due to the nature of this observational study, which was conducted through a phone call, we have missed several factors that might influence the pharmacists’ responses, including the pharmacists’ sociodemographic data, pharmacy turnover, the number of staff per shift, and the socioeconomic status of patients served by their corresponding pharmacies. Nonetheless, this is an observational study that aimed to identify the spontaneous responses of the pharmacists’ and did not intend to study the causality of these responses. Furthermore, only one simulated patient was utilized for documenting several observations at a time, which might subject the study to recall bias. However, the simulated patient was instructed to complete the observation checklist during or immediately after each call to reduce the underreporting of events.

Moreover, additional limitations may have played a role in this study. The SP called the pharmacies and asked for immediate help regarding her symptoms. Perhaps it would have been better initially to ask the pharmacist if the time was right and to request an appointment if the pharmacist was busy. This could have limited the number of non-respondents (14 %). Nonetheless, we intended to investigate how pharmacists were ready to handle sudden phone calls, and how to respond to them. Besides, we used regular phone calls instead of video calls, where the SP and the pharmacists were not able to have visual communication and observe facial expressions that may have had an impact on the interaction. Furthermore, the SP is an unknown client to the contacted pharmacists, which might have negatively influenced their responses and provision of pharmaceutical care through telemedicine, especially since it is an unpaid service.

## Conclusions

 This study revealed the actual implementation and quality of telehealth services provided by the community pharmacists during the COVID-19 pandemic. An unsatisfactory level of preparedness through means of telehealth technology was evident. This caused the quality of pharmaceutical care services provided to high-risk patients via telehealth to be below expectations. Therefore, in light of these findings, healthcare authorities should encourage community pharmacists to adopt telehealth technologies, guide them to gain the necessary skills, and to recognize their extra efforts with financial compensations. These means are crucial to optimize pharmacists’ effective engagement in telehealth and improve the overall healthcare outcomes of patients.

## Supplementary Information

**Additional file 1.**

## Data Availability

The dataset presented in this article is available only upon reasonable request, since it contains confidential information. Requests to access the datasets should be directed to the first author (r.itani@bau.edu.lb).
