# Supplementary material for: Provision of pharmaceutical care to suspected high-risk COVID-19 patients through telehealth: a nationwide simulated patient study
Source: BMC Health Serv Res. 2021 Sep 21;21:997. doi: 10.1186/s12913-021-07014-x (PMC8454989; doi:10.1186/s12913-021-07014-x)
Supplement: Supplementary file 1 — Additional file 1. [file 12913_2021_7014_MOESM1_ESM.docx]

***Supplementary Appendix***

**Provision of Pharmaceutical Care to Suspected High-Risk COVID-19 Patients through Telehealth: A Nationwide Simulated Patient Study**

**Data Collection Form**

**Form Code: ______**

**Visiting Information:**

| **Day** | **Month** | **Year** |  | **Hour** | **Minutes** | **Time** |  | **Call duration (minutes)** |
| --- | --- | --- | --- | --- | --- | --- | --- | --- |
|  |  | **2021** |  |  |  | **⭘ AM ⭘ PM** |  |  |

**Sociodemographic Characteristics of the Contacted Pharmacies**

**Pharmacy Province:** ○ Beirut ○ Mount Lebanon ○ North ○ South ○ Bekaa

**Sex:** ○ Male ○ Female

**Relevant medical data asked by the contacted pharmacist**

1. **Patient Information**

 Age

 Pregnancy/lactation

 Occupation (to rule out high-risk occupations, such as HCP, LTCF)

 Residence and members sharing living

 Duration of DM

 FBG and RBG readings

 Frequency of routine DM clinic visits

 Chronic medical condition(s)

 Chronic medication(s)

 Adherence to diet and physical activity

 DM was controlled/uncontrolled before the onset of new symptoms

 Presence of fruity-smelling breath

 Insulin doses were constant/fluctuating before the onset of new symptoms

 None of the above

1. **Assessment of COVID-19 Exposure**

 Close contact with someone with symptoms, diagnosed OR tested positive for COVID-19, in two weeks before feeling sick

*Close contact (within 6 feet of infected person for a cumulative total of 15 minutes or more over a 24-hour period)

 Travel history

Attending a social gathering, or was in crowded indoor settings (with more than 10 people without universal mask wearing and/or physical distancing)

 None of the above

1. **Assessment of COVID-19 related symptoms and treatments**

 Nature of symptoms of COVID-19 (cough, shortness of breath or difficulty breathing, fever or chills, muscle or body aches, new loss of taste or smell, vomiting or diarrhea)

 Onset & duration of symptoms (When did the symptoms start?)

 Life-threatening symptoms (red flag symptoms, such as severe dyspnea, persistent chest pain, new confusion, inability to wake up or stay awake, bluish lips or face)

 Medications used to manage symptoms (Have you tried any treatment?)

 History of Flu vaccination

 None of the above

 Others, ­­­­____________________________________________________________________________

**Pharmacist Responses to the patient’s situation (Select all that apply):**

**First Response:**

 Advised to seek medical attention since she is a high-risk patient

 Advised to do a PCR test

 None of the above

**Medical Attention:**

○ Advised to go to the Emergency Room (ER)

○ Advised to contact her healthcare provider

○ Referred to a specific healthcare facility, e.g. private clinic having a contract with the pharmacy

**Other Responses:**

 Asked to end the call since he/she is busy and can’t help

 Informed the patient that she MIGHT have COVID-19

 Assured/confirmed that she has COVID-19

 Assured that this is NOT COVID-19 and that this is flu

 Mentioned that the PCR test is not necessary in her condition

 Advised the patient to isolate herself for 14 days

 Recommended to avoid contacting others (especially her mother)

 Recommended an antipyretic for fever

 Recommended other medication(s)

 Recommended a supplement

 Recommended non-pharmacological measure(s)

 Advised to stick to PPE (mask, disinfectant, gloves…)

 Advised to monitor red-flag symptoms

 Advised to monitor her blood glucose

 Emphasized on the importance of avoiding the use of antimicrobials (antibiotics, antivirals) without prescription

 Avoid sugar intake

 Increase insulin dose

 None of the above

 Other: _____________________________________________________________________________

**Insulin (brand name, generic name, dose, dosage regimen, duration):**

______________________________________________________________________________

**Medications prescribed:**

 Antibiotic
 Antiviral
 Antihistamine
 Pseudoephedrine
 Corticosteroids

**Supplements prescribed**

 Zinc

 Vitamin C
 Vitamin D
 Multi-vitamins

**First medication (Name, dosage form, dose, dosage regimen, duration):**

______________________________________________________________________________

**Second medication (Name, dosage form, dose, dosage regimen, duration):**

______________________________________________________________________________

**Third medication (Name, dosage form, dose, dosage regimen, duration):**

______________________________________________________________________________

**Non-pharmacological measures:**

______________________________________________________________________________

**Assessment of the Pharmacist’s Communication Skills**

| **General Communication Skills** | **Yes** | **No** | **NA** |
| --- | --- | --- | --- |
| 1. Introduced him/herself |  |  |  |
| 1. Solicited the patient’s agenda (How can I help you?) |  |  |  |
| 1. Used proper and clear language (No jargon) |  |  |  |
| 1. Showed empathy (demonstrated sincere interest in the patient’s emotional needs) |  |  |  |
| 1. Demonstrated active listening (provided feedback, briefly summarized his/her understanding to clarify any message) |  |  |  |
| 1. Gave appropriate closure (summarized the patient’s concern and asked if she had any other questions) |  |  |  |
